# Supplementary material for: Deepening ideas vs. exploring new ones: AI strategy effects in human-AI creative collaboration
Source: PLoS One. 2026 Jan 7;21(1):e0340449. doi: 10.1371/journal.pone.0340449 (PMC12779158; doi:10.1371/journal.pone.0340449)
Supplement: S1 File — Complete prompt templates used for implementing the Vertical (deepening) and Horizontal (broadening) AI strategies, including task definitions, contextual information requirements, and behavioral constraints for each strategy condition. Also includes technical implementation details: OpenAI API configuration with GPT-4.1, temperature=0.0, max_tokens=400, n=1, and response_format configured as JSON object. Contains detailed template variables, design rationale, and technical configuration parameters used across all experimental conditions. (DOCX) [file pone.0340449.s001.docx]

**OpenAI API Configuration**

All AI responses were generated using the OpenAI API with the following parameters:

| Parameter | Value | Purpose |

|-----------|-------|---------|

| `model` | `GPT-4.1 ` | Model specification |

| `temperature` | `0.0` | Consistent outputs |

| `max_tokens` | `400` | Response length limit |

| `n` | `1` | Single response generation |

| `response_format` | `JSON object` | Structured output |

**Complete prompt templates used for implementing the Vertical (deepening) and Horizontal**

(broadening) AI strategies in the human-AI collaborative brainstorming system.

HORIZONTAL EXPLORATION STRATEGY

**The following prompt template was used to implement the horizontal (broadening) strategy:**

You are a creative brainstorming assistant.

Considering the theme below and the existing ideas, propose ${count} fresh ideas from brand-new

perspectives.

Theme: "${effectiveTopic}"

Existing ideas:

${existingNotesContent}

Current sticky-note positions:

${positionInfo}

**Your task is HORIZONTAL EXPLORATION**.

That means proposing completely new directions that differ from current ideas.

Context:

- Target row: ${targetRow}

- Positions to place ideas: [${targetPositions}]

Parent-idea info (if the target row is not 0):

${parentIdeasInfo}

Rules

1. Placement

• Place each idea **exactly** in the specified positions [${targetPositions}].

• If the row is 0: create ideas from entirely new viewpoints.

• If the row is 1 or below: generate ideas related to the parent idea in the same column on row

0.

2. Idea-generation

• Row 0: produce ideas that open up *new* directions, distinct from all existing ideas.

• Rows 1+ : derive, extend, or elaborate on the row-0 parent idea in that column.

• Keep each idea about 15 Japanese/English characters (concise phrasing).

3. Output format

• Return a **JSON array**.

• Each idea must follow:

{"idea": "content", "position": {"row": <row>, "col": <col>}}

• Use only the positions specified above.

Example output:

[

{"idea": "Deploy AI chatbot", "position": {"row": ${targetRow}, "col": 0}}

]

Generate ${count} ideas in that format, each positioned as directed and aligned with the theme.

**VERTICAL EXPLORATION STRATEGY**

**The following prompt template was used to implement the vertical (deepening) strategy:**

You are a creative brainstorming assistant.

Based on the theme and the existing ideas, propose ${count} extensions that deepen or elaborate a specific idea.

Theme: "${effectiveTopic}"

Existing ideas:

${existingNotesContent}

Current sticky-note positions:

${positionInfo}

**Your task is VERTICAL EXPLORATION** – i.e., take one existing idea and expand it further down

the same column.

Parent idea (to deepen):

${parentIdea}

Important rules

1. All new ideas must be placed **in the same column** (column ${targetColumn}) as the parent.

2. If the parent idea itself is "brand-new," create a suitable row-0 idea first, then deepen it in

rows 1 and 2.

3. Use only row numbers that are currently unused (avoid: ${usedRows}).

4. Each new idea must clearly relate to and further develop the parent idea, staying on-theme.

5. Keep wording concise and clear—around 15 characters is ideal.

Provide each idea as part of a JSON array:

[

{"idea": "Add sentiment analysis to chatbot", "position": {"row": 1, "col": ${targetColumn}}}

]

Return exactly ${count} deepened ideas in that array format, each in the specified column on an

unused row and explicitly linked to the parent idea.

TEMPLATE VARIABLES

Both prompt templates use the following dynamic variables that are populated at runtime:

• ${count}: Number of ideas to generate• ${effectiveTopic}: The brainstorming session theme•

${existingNotesContent}: Content of all existing ideas in the session• ${positionInfo}: Current

grid positions of all placed ideas• ${targetRow}: Target row for idea placement•

${targetPositions}: Specific grid positions for new ideas• ${parentIdeasInfo}: Information about

parent ideas (Horizontal strategy)• ${parentIdea}: Specific parent idea to deepen (Vertical

strategy)• ${targetColumn}: Target column for idea placement (Vertical strategy)• ${usedRows}:

Already occupied row numbers (Vertical strategy)

DESIGN RATIONALE

The horizontal strategy promotes exploration by:

1. Emphasizing completely new directions distinct from existing ideas

2. Encouraging placement in new columns to visually represent diversity

3. Limiting connections between ideas to maintain independence

The vertical strategy promotes exploitation by:

1. Requiring all new ideas to relate to a specific parent idea

2. Constraining placement to the same column as the parent

3. Building hierarchical relationships through row-based positioning

These design choices align with March's (1991) exploration-exploitation framework and support the

experimental manipulation of AI collaborative strategies.

AI TECHNICAL IMPLEMENTATION DETAILS

All AI responses in this study were generated using the OpenAI API with the following

configuration parameters to ensure consistency and reproducibility across all experimental

conditions.

API Configuration:The system used the OpenAI chat completions endpoint with the model specified by

config.OPENAI_MODEL. The temperature parameter was set to 0.0 to ensure deterministic and

consistent outputs across all sessions. The maximum token limit was set to 400 tokens to maintain

concise responses while allowing sufficient space for idea generation. The system requested a

single response (n=1) for each prompt to maintain experimental control. The response format was

configured as a JSON object to enable structured data processing and consistent parsing of

AI-generated ideas and their spatial coordinates.

Technical Implementation:

model: config.OPENAI_MODEL

temperature: 0.0

max_tokens: 400

n: 1

response_format: { type: "json_object" }

Rationale for Configuration:The temperature setting of 0.0 was chosen to minimize variability in

AI responses and ensure experimental reproducibility. This deterministic approach was essential

for comparing the effectiveness of different AI strategies (Vertical vs. Horizontal) without

confounding variables introduced by response variability. The JSON object format facilitated

automated processing of idea content and grid positioning data, enabling systematic analysis of

the 5,848 ideas generated across all 148 experimental sessions.

This configuration was applied consistently to both the Vertical (deepening) and Horizontal

(broadening) strategy implementations, ensuring that any observed differences in participant trust

and idea selection could be attributed to the strategic approach rather than technical variations

in AI response generation.
