# Supplementary material for: Deepening ideas vs. exploring new ones: AI strategy effects in human-AI creative collaboration
Source: PLoS One. 2026 Jan 7;21(1):e0340449. doi: 10.1371/journal.pone.0340449 (PMC12779158; doi:10.1371/journal.pone.0340449)
Supplement: S2 File — Comprehensive user instructions for the frontend interface, including video explanations, screen-by-screen interface descriptions, and complete questionnaire items. Contains detailed instructions for brainstorming session operations, AI behavior evaluation scales (GQS and MDMT), and all experimental materials used for participant guidance throughout the study. (DOCX) [file pone.0340449.s002.docx]

# User Instructions for Frontend Interface(translated in English)

**## Video Instructions(translated in English)**

This experiment involves AI and humans collaborating to generate ideas efficiently through alternating turns.At the top of the screen, you can see the current theme displayed. The upper right shows the current turn progress and an "End Turn" button. You can also see the user turn and AI turn counters displayed in the upper right corner.When the turn count reaches the set number, the session will end.

Let's look at how to actually use this tool. In this system, you and the AI take turns generating ideas.The green section in the center of the screen displays "It's your turn. Please write your ideas on sticky notes." In each turn, you can place one sticky note, after which the turn ends.To add a sticky note, click on the position where you want to add it.

Once a sticky note is added, write your idea on it. For example, you might input an idea like "Improve meeting minutes."

After entering your idea, click the "End Turn" button. Please note that if the idea is empty, you cannot end the turn. This will then move to the AI's turn, where the AI will automatically generate ideas.

This tool arranges ideas on a grid. The grid is an important feature that can express vertical and horizontal relationships. New category ideas should be placed in new columns. For example, "Introduction of AI facilitator" is a completely different idea, so it should be added to a separate column. On the other hand, if you want to delve deeper into existing ideas, place them vertically connected below that idea. For example, "Having meetings while standing in a cafe" is an idea inspired by existing ideas, so it should be placed below the "Encourage standing and walking" idea.

This makes the relationships between ideas visually clear.

You may want to move sticky notes after placing them. In that case, you first need to delete the old sticky note. To delete, click the × button in the upper right of the sticky note.

After deleting, you can add a sticky note to a new position again.

Also, this idea space may extend beyond the currently visible range. Please refer to the minimap in the lower right to see where you currently are.

This concludes the explanation. After this, you will move to a test session where you can freely add, edit, and delete sticky notes.

Once you get familiar with the test session, there will be a button to move to the actual session, so please proceed there.

After completing the actual session and answering the questionnaire, the experiment will be finished.

**## 1. Initial State Screen (initial)**


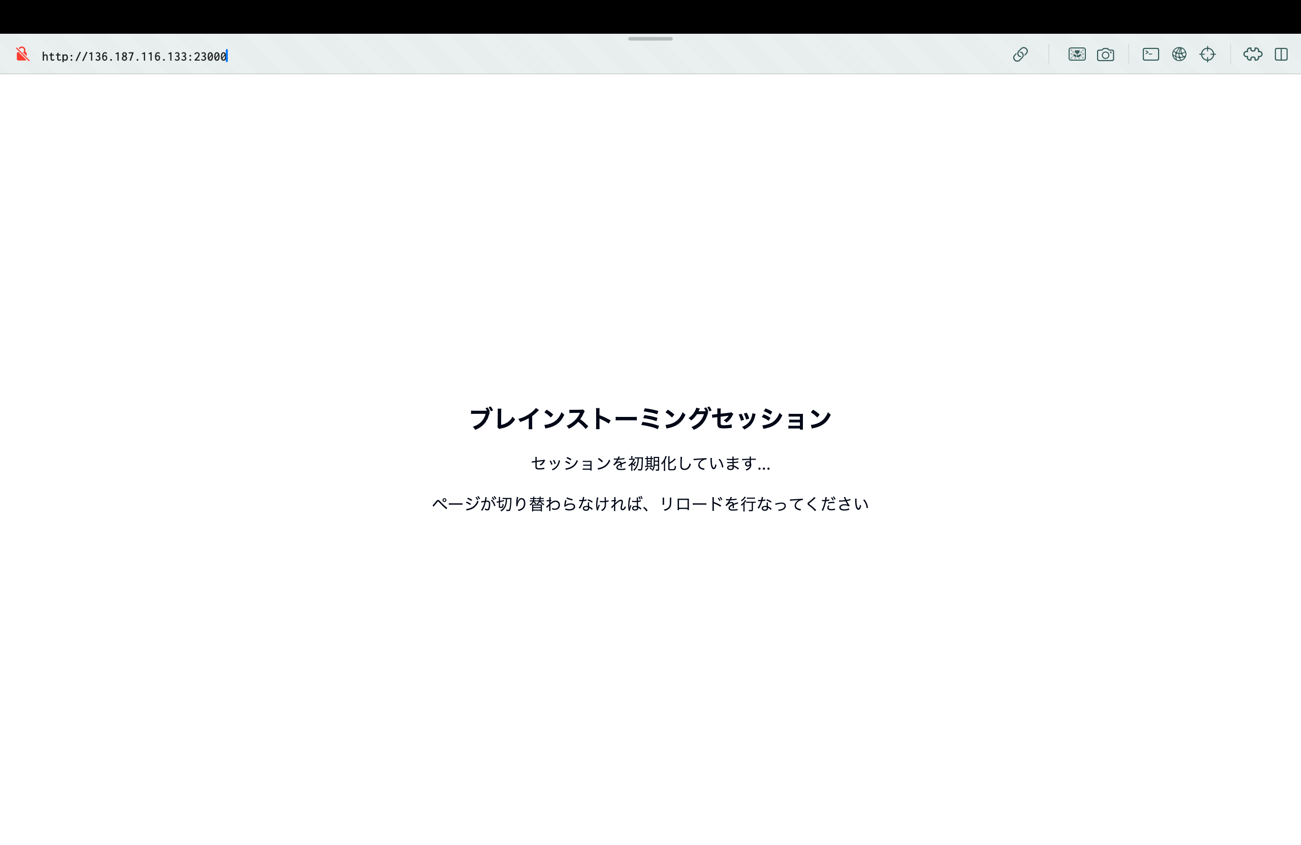


```

Brainstorming Session

Initializing session...

If the page does not switch, please reload.

```

**## 2. Video Explanation Screen (video)**


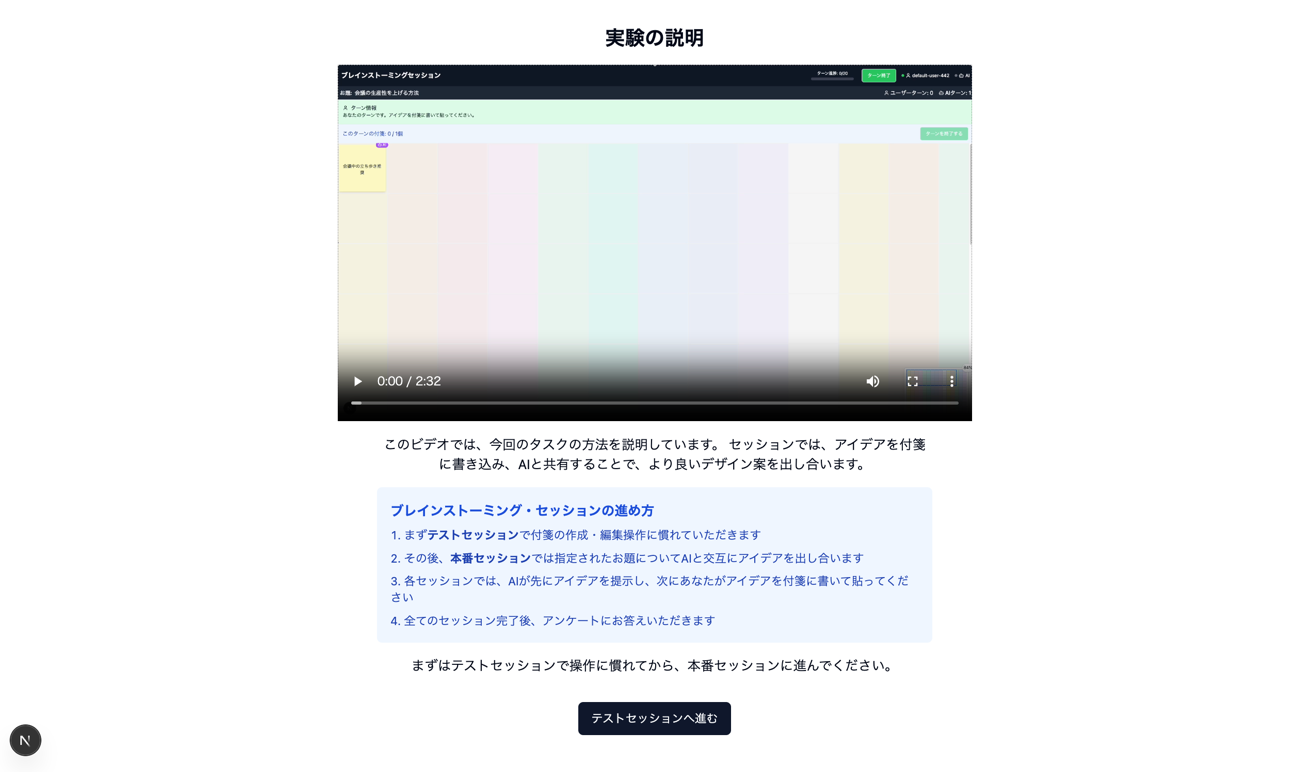


### Title

```

Experiment Explanation

```

### Description

```

This video explains the method for today's task.

In the session, you will write ideas on sticky notes and share them with AI to generate better design ideas together.

```

### Process Explanation

```

How to Proceed with Brainstorming Sessions

1. First, familiarize yourself with creating and editing sticky notes in the test session

2. Then, in the actual sessions, you will alternate with AI to generate ideas on specified topics

3. In each session, AI will present ideas first, then you will write and post your ideas on sticky notes

4. After completing all sessions, please answer the questionnaire

```

### Important Notes

```

Please familiarize yourself with the operations in the test session before proceeding to the actual session.

```

### Confirmation Modal

```

Confirmation

Did you watch the explanation video? If not, please go back and watch the video.

```

**## 3. Test Session Screen (test)**


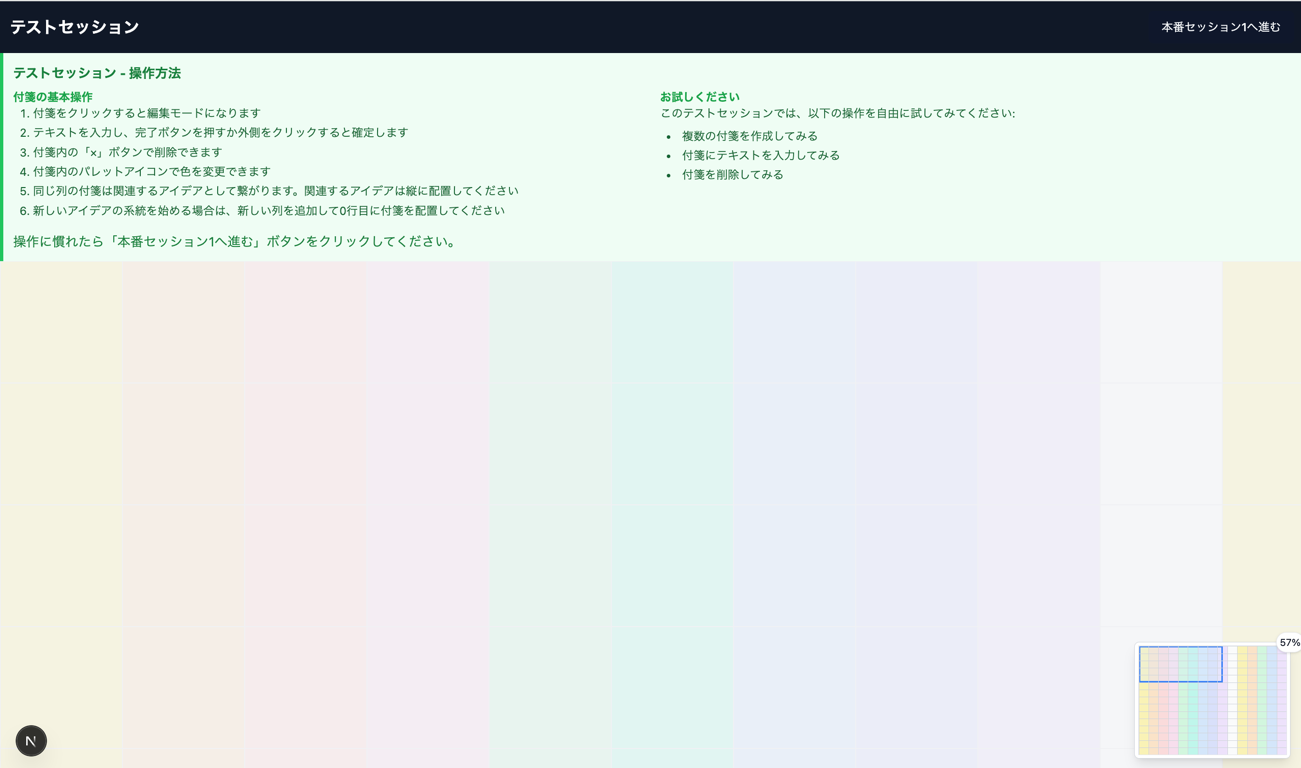


### Header

```

Test Session

```

### Operation Instructions

```

Test Session - How to Operate

Basic Sticky Note Operations:

1. Click on a sticky note to enter edit mode

2. Enter text and press the complete button or click outside to confirm

3. Delete using the "×" button within the sticky note

4. Change color using the palette icon within the sticky note

5. Sticky notes in the same column are connected as related ideas. Please arrange related ideas vertically

6. To start a new idea category, add a new column and place a sticky note in row 0

Please Try:

In this test session, please freely try the following operations:

• Create multiple sticky notes

• Enter text in sticky notes

• Delete sticky notes

Once you're comfortable with the operations, click the "Proceed to Session 1" button.

```

**## 4. Topic Introduction Screens**


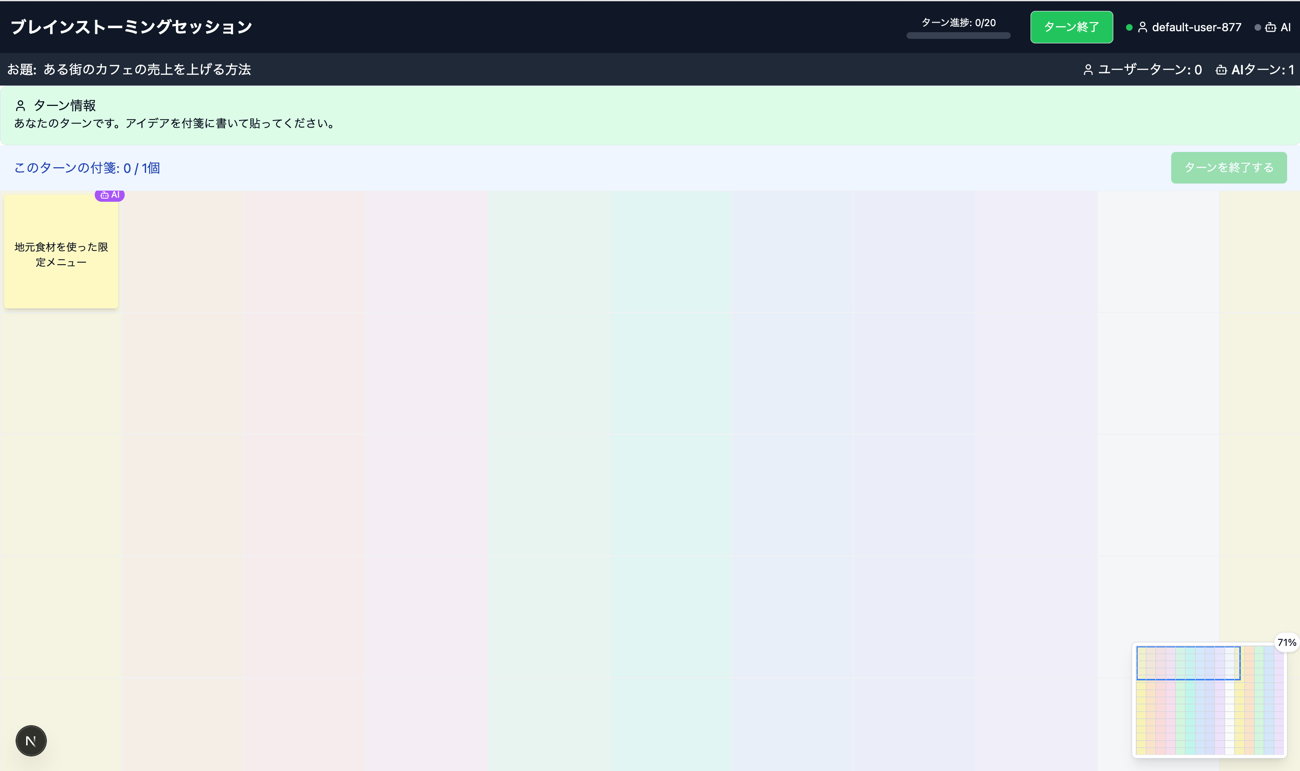


### Session Topic

```

How to Increase Sales of a Local Cafe

Please think of ideas to improve the sales of a typical cafe in your town. Let's brainstorm from multiple perspectives including new services, customer experience improvements, and marketing strategies.

Example Points to Consider:

• Ideas for improving store environment and interior

• Proposals for new menus and products

• Mechanisms to increase customer return rates

• Digital utilization for customer acquisition

Hint:

Thinking about your favorite cafe or ideal cafe experience will help you come up with more specific ideas.

Consider various aspects such as cafe atmosphere, services, and products.

```

## 5. Brainstorming Session Screens (session1, session2)

### User Turn Display

```

Sticky notes this turn: X / Y

(With content: Z / Required: 1)

※This turn's sticky notes are at the limit. Please edit or delete.

or

※Delete to add new sticky notes

```

### Turn End Restrictions

```

In this turn, you can only place up to Y sticky notes. Please edit or delete existing sticky notes before adding more.

```

```

To end the turn, at least one sticky note you added this turn must have content.

```

## 6. Idea Evaluation Screens (evaluate1)


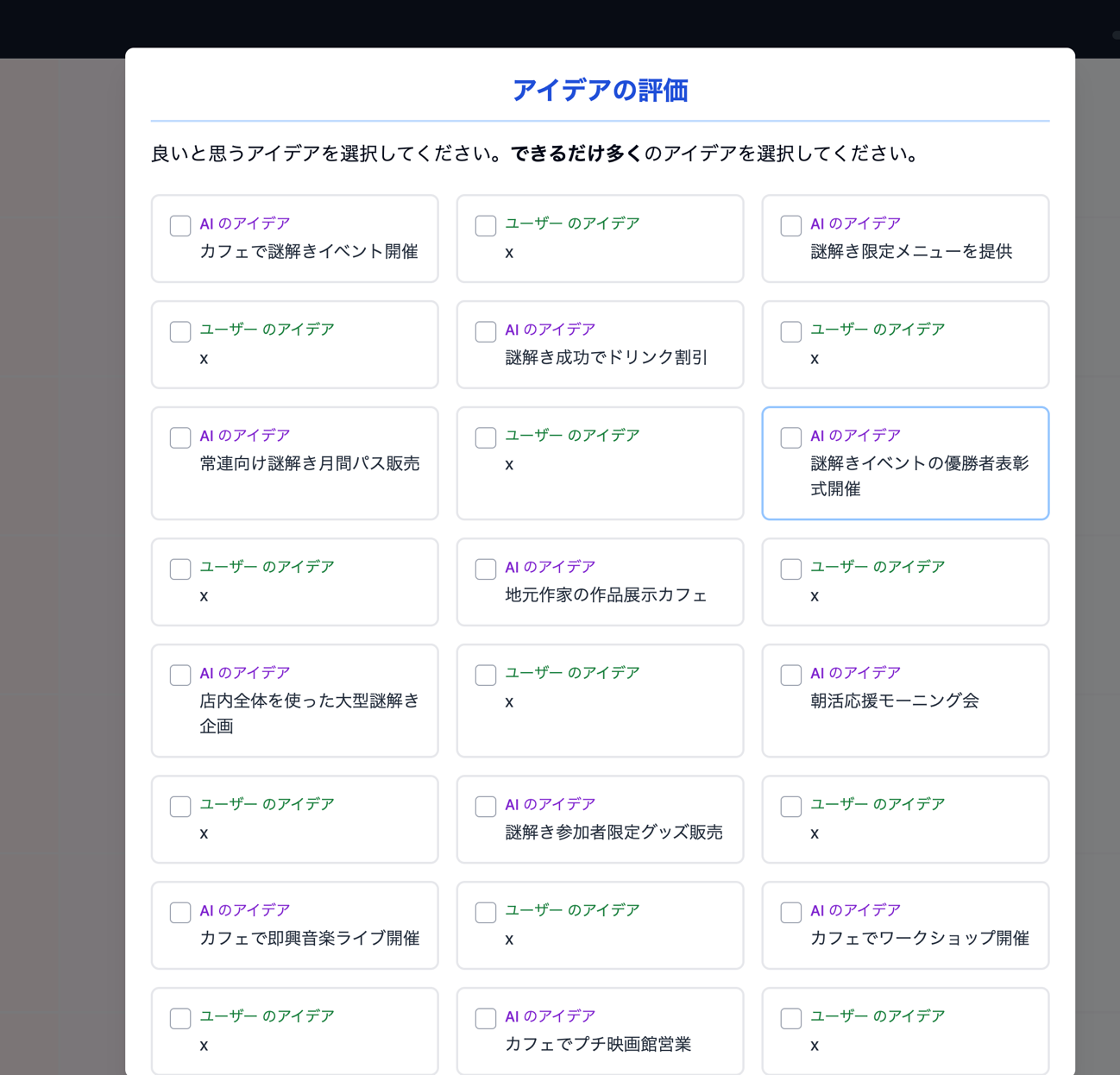


```

Idea Evaluation

Please select ideas you think are good. Please select as many ideas as possible.

```

## 7. Questionnaire Screens

### Section Instructions

#### Participant Information

```

In this section, we ask for basic participant information for statistical analysis.

Age* (Required)

Gender* (Required): Male / Female / Other

```

#### AI Feedback

```

In this section, we ask about AI's behavior and contributions during brainstorming.

Please select the option that best describes AI's behavior during brainstorming*

Options:

1. Could not identify characteristics of AI's behavior

- Could not find clear patterns in AI's behavior or contributions

2. Specialized in deepening existing ideas

- AI mainly focused on developing or elaborating on ideas that I or other participants had proposed

3. Specialized in creating new ideas

- AI mainly focused on proposing new perspectives or completely different ideas

Please freely comment on AI's contributions (Optional)

```

#### AI Evaluation (GQS)

```

In the following questions, we ask about the AI you used for idea generation.

Please evaluate each item on a 5-point scale indicating which adjective better applies.

Anthropomorphism:

1. Machine-like ←→ Human-like

2. Artificial ←→ Natural

3. Unconscious ←→ Conscious

4. Still ←→ Lively

5. Mechanical ←→ Organic

Animacy:

1. Dead ←→ Alive

2. Stagnant ←→ Lively

3. Inactive ←→ Active

4. Unresponsive ←→ Responsive

5. Sleepy ←→ Awake

Likeability:

1. Unpleasant ←→ Pleasant

2. Unkind ←→ Kind

3. Unpleasant ←→ Pleasant

4. Awful ←→ Nice

5. Scary ←→ Not scary

Perceived Intelligence:

1. Incompetent ←→ Competent

2. Unresponsive ←→ Responsive

3. Ignorant ←→ Knowledgeable

4. Irresponsible ←→ Responsible

5. Unintelligent ←→ Intelligent

Perceived Safety:

1. Anxious ←→ Calm

2. Calm ←→ Agitated (reversed item)

3. Peaceful ←→ Surprised (reversed item)

```

#### Trust Evaluation (MDMT)

```

In this section, we evaluate trust in the AI you used for brainstorming.

Please evaluate the following items on a scale from 0 (not at all) to 7 (very much so).

Reliable:

1. Reliable

2. Predictable

3. Dependable

4. Consistent

Capable:

1. Capable

2. Skilled

3. Competent

4. Meticulous

Ethical:

1. Ethical

2. Respectable

3. Has integrity

4. Honest/upright

Sincere:

1. Sincere

2. Genuine/authentic

3. Straightforward

4. Real/trustworthy

```

### Error Messages

```

X questions are unanswered. Please answer all questions before proceeding.

```

## 8. Completion Screens (survey, finish)

### Survey Response Screen

```

Session Complete

The brainstorming session has been completed.

Please proceed to answer the questionnaire.

Your ID: [ID Display]

This ID is required when answering the questionnaire. Please take notes or capture the screen.

```

### Final Completion Screen

```

The task is now complete.

Please return to the Yahoo CrowdSourcing page and enter the following keyword:

Keyword: brain

※This keyword is necessary for task completion, so please be sure to copy it.

```
