# Supplementary material for: Deepening ideas vs. exploring new ones: AI strategy effects in human-AI creative collaboration
Source: PLoS One. 2026 Jan 7;21(1):e0340449. doi: 10.1371/journal.pone.0340449 (PMC12779158; doi:10.1371/journal.pone.0340449)
Supplement: Attachment — Submitted filename: Response to Reviewers.docx [file pone.0340449.s004.docx]

Manuscript ID: PONE-D-25-40481

Title: Deepening Ideas vs. Exploring New Ones: AI Strategy Effects in Human-AI Creative Collaboration

Journal: PLOS ONE

Dear Dr. Md. Rabiul Awal and Reviewers,

Thank you for the opportunity to submit a revised version of our manuscript. We are grateful for your guidance and for the reviewers’ thoughtful and constructive feedback. We have carefully revised the manuscript and provide below a point-by-point response. For ease of checking, page and line numbers refer to the revised clean manuscript. We have also uploaded the following files: the Response to Reviewers, the Revised Manuscript with Track Changes, and the Clean Manuscript.

Best regards,

### *Journal Requirements*

1. Please ensure that your manuscript meets PLOS ONE's style requirements, including those for file naming. The PLOS ONE style templates can be found at https://journals.plos.org/plosone/s/file?id=wjVg/PLOSOne_formatting_sample_main_body.pdf and https://journals.plos.org/plosone/s/file?id=ba62/PLOSOne_formatting_sample_title_authors_affiliations.pdf

We appreciate the observation. The manuscript has been reformatted to comply with the PLOS ONE templates (main body; title/authors/affiliations). All submission files follow the required naming conventions. Figure files are provided individually and were pre‑checked with PACE.

2. Please note that PLOS One has specific guidelines on code sharing for submissions in which author-generated code underpins the findings in the manuscript. In these cases, we expect all author-generated code to be made available without restrictions upon publication of the work. Please review our guidelines at https://journals.plos.org/plosone/s/materials-and-software-sharing#loc-sharing-code and ensure that your code is shared in a way that follows best practice and facilitates reproducibility and reuse.

Thank you for highlighting this observation.

The code will be made **publicly available without restriction upon publication** and archived with a **DOI**. We will add the final **URL/DOI during production/author proofs**.

3. Thank you for stating the following financial disclosure:

[JST, CREST (JPMJCR21D4), Japan.].

Please state what role the funders took in the study. If the funders had no role, please state: "The funders had no role in study design, data collection and analysis, decision to publish, or preparation of the manuscript."

If this statement is not correct you must amend it as needed.

Please include this amended Role of Funder statement in your cover letter; we will change the online submission form on your behalf.

"The funders had no role in study design, data collection and analysis, decision to publish, or preparation of the manuscript."

Thank you for bringing this to our attention.

We will include the final Role of Funder statement in the cover letter as requested.

4. If the reviewer comments include a recommendation to cite specific previously published works, please review and evaluate these publications to determine whether they are relevant and should be cited. There is no requirement to cite these works unless the editor has indicated otherwise.

We appreciate the observation.

We reviewed suggested publications and cited only those directly relevant to our research questions and interpretations.

5. Please review your reference list to ensure that it is complete and correct. If you have cited papers that have been retracted, please include the rationale for doing so in the manuscript text, or remove these references and replace them with relevant current references. Any changes to the reference list should be mentioned in the rebuttal letter that accompanies your revised manuscript. If you need to cite a retracted article, indicate the article’s retracted status in the References list and also include a citation and full reference for the retraction notice.

Thank you for bringing this to our attention.

We audited the reference list for completeness, accuracy, and retraction status. No retracted items are presently cited.

In addition, to strengthen the Limitations section and to justify our notes on domain-specificity and cross-cultural considerations, we added two references to the reference list and cite them in the revised manuscript (clean p. 20, lines ~741 and ~751–753): Baer (1998), Creativity Research Journal, 11(2), 173–177, doi:10.1207/s15326934crj1102_7; and Niu & Sternberg (2001), International Journal of Psychology, 36(4), 225–241, doi:10.1080/00207590143000036. These additions clarify interpretation; they do not change our results or conclusions.

### *Response to Reviewer #1*

We sincerely thank the reviewer for the time, careful reading, and constructive comments. We have revised the manuscript accordingly and indicate specific changes and locations below.

*“****The scope of the study is limited. It only focuses on a specific task (brainstorming for increasing cafe sales), which might not generalize to other creative tasks or contexts. No tests for the implications for varied domains****.”*

We sincerely thank the reviewer for their constructive feedback regarding the study’s scope. The reviewer rightly points out the need for caution when considering generalizability. Our initial decision to focus on a single theme (increasing café sales) was driven by the goal of examining effects within a controlled, single-session experiment while minimizing participant burden.

To address this important point, we have made two key revisions. First, we have specified the task in the Abstract to ensure the study’s scope is clear from the outset. Second, while we had previously touched upon this in the Limitations section, we have now substantially strengthened this point by citing prior research indicating that creativity is task-dependent (p. 20, lines 738–740) and added a new citation [56] (Baer, 1998). We hope these revisions adequately address the reviewer's concern.

*“****No long-term effects are addressed; only the experiment is conducted in a single session, limiting insights into the long-term dynamics of human-AI collaboration. Long-term effects and the evolution of trust over time are not addressed.****”*

We thank the reviewer for this crucial point regarding the long-term effects. We agree that the single-session design is an important limitation, stemming from our choice of a web-based experiment for data collection.

To address this, we have revised the Limitations and Future Work section to explicitly state this constraint. Furthermore, we have strengthened the discussion by citing prior research on trust dynamics in human-AI relationships, and we have clearly identified multi-session validation as a key avenue for future research (p. 20, lines 734–735). We hope this clarifies the scope of our current findings and addresses the reviewer’s valuable concern.

*“****Only homogeneous culture is discussed; no cross-cultural differences are discussed****.”*

We are grateful to the reviewer for raising the important issue of cultural homogeneity. While our original manuscript acknowledged that our sample was drawn from Japan, we concur that the discussion of this limitation lacked sufficient grounding.

In response, we have revised the Limitations and Future Work section to more strongly support this point. Specifically, we have now cited prior research([57], Niu & Sternberg, 2001) demonstrating cultural differences in creative evaluation (e.g., U.S. vs. China) to provide a clearer basis for why this is a significant consideration (p. 20, lines 749–751). We believe this revision now more thoroughly addresses the reviewer’s valid concern.

### Response to Reviewer #2

We are deeply grateful to Reviewer #2 for the careful reading and positive evaluation. In particular, we appreciate your recognition of our positioning of human-AI co-creativity and the supportive role of GPT-4.1, as well as your assessment that the exploration-exploitation framework appropriately serves our aim of comparing exploring new ideas and deepening existing ideas using trust in the AI collaborator and idea adoption as outcome measures. We have revised the manuscript accordingly and indicate specific changes and locations below.

***Line 270: “Each cell in the 15×15 grid space can contain a maximum of one idea (in a sticky note format). This constraint is intended to encourage participants to engage in strategic spatial placement and stimulate creative thinking”.***

***Participants are encouraged to do so, but were they also explicitly instructed to do strategic planning? Were they given examples and able to get familiar with the design through a mock example?***

Thank you for your valuable feedback. The question of whether participants received explicit instructions on strategic placement is critical for clarifying our experimental design, and we appreciate you raising this point.

To address this, we have revised the “Experimental Procedure” section (p. 10, lines 362–367). As the updated manuscript now clarifies, participants were not merely encouraged to engage in strategic placement; they received explicit instructions with concrete examples during a practice session before the main experiment.

As detailed in the Supporting Information (S2 File), a video tutorial explained strategic placement methods, such as “placing new ideas in new columns (horizontal exploration)” and “deepening related ideas by connecting them vertically (vertical deepening).” Afterward, participants entered a “test session” where they could freely practice these operations, ensuring they were familiar with both the interface and the strategic placement approach before the formal experiment began.

We believe this revision makes it clear that participants operated under well-instructed and controlled conditions. We thank you again for your insightful comment, which has helped us improve the clarity of our manuscript.

***Lines 280-281: “In the experiment, the human and the AI each generated 20 ideas, creating a total of up to 40 ideas.”***

***Did they have to come up with exactly 20 ideas each? Or if they ran out of ideas before that they could finish early? Was there a time limit to how long they were allowed to think for each idea? It sounds as if they were required to write exactly 20 ideas (as it is also mentioned that their progress is tracked on a progress bar), but then saying “a total of up to 40 ideas” sounds as if it could be less than 40 ideas. Same at line 383: “maximum total of 40 ideas” which sounds as if it could be less.***

Thank you very much for your feedback, and we apologize for the lack of clarity in our description. To clarify, participants and the AI were equally given 20 “opportunities” or turns to generate ideas, and all participants completed the full 40 turns without the option to finish early.

There was no time limit for thinking about each idea; the progress bar was simply meant to show the progress through the turns.

As you correctly pointed out, the final number of ideas could be less than 40. This is because the system was designed to allow participants to freely delete or overwrite their own ideas to correct mistakes or refine their thoughts. We believe that the ideas ultimately left by the participants are those they judged to be more important and carefully selected, and thus, this functionality does not negatively impact the quality of the final idea selection.

Since this mechanism was unclear in the manuscript, we have now added a sentence to the “System Overview and Architecture” section for clarification (p. 7, lines 264–265). We believe this revision resolves the ambiguity in the experimental procedure and are grateful for your valuable feedback, which has helped us improve the clarity of our manuscript.

***Line 309: In the deepening-ideas condition it says “the parent idea to be deepened”. Does the parent idea only refer to the human original idea? Or can the AI also deepen a response that it gave by itself in response to the human original idea? If the human participant wrote a new idea in response to the AI deepened idea, will the AI deepen the first original human idea further or switch to the new human idea and deepen or expand this further?***

***Also, it is a valid choice to stop deepening an idea after 9 instances of an expansion - but does the limit of 9 refer to the number of answers the AI can give or in total? So if the humans themselves have already written 5 ideas in that specific column, will the AI only deepen this category 4 more times, or another 9 times?***

We sincerely thank the reviewer for their insightful questions. Your points helped us identify ambiguities in the description of the AI’s behavior, and we have revised the manuscript to be more precise.

First, to clarify the selection logic for the “parent idea,” the AI does not dynamically respond to the user’s latest input. Instead, it follows a deterministic rule, as detailed in Algorithm 1: it scans the grid from left to right and selects the first column with available capacity to deepen. In this process, it does not matter whether the parent idea was created by the human or the AI. We have revised the “AI Strategy Implementation” section to clarify this point (p. 7, lines 274–276).

Next, regarding the deepening limit, the cap of nine ideas per column refers to the combined total of ideas generated by both the human and the AI (i.e., a maximum of nine ideas per column, combined human + AI). Therefore, if a human has already placed five ideas in a column, the AI will only add four more. We have also amended the text to make this clear to the reader (p. 7, lines 286–288).

Thank you again for your valuable feedback, which has significantly helped improve the clarity and reproducibility of our methodology.

***Line 543: “This study empirically validated two AI strategies”. Can you really say that the study “validated” these two strategies? Validation means that both strategies were proven as being correct, but the study at hand rather assesses and compares both strategies. “Validated” carries a very strong connotation of correctness which is not the research question here.***

We thank the reviewer for their precise and insightful feedback. You are correct that the word “validated” carries a strong connotation of proving the correctness of the strategies, which was not the intent of our comparative study. We agree that this was an inappropriate term.

To address this, we have revised the first sentence of the Discussion section (p. 15, line 533). The term “validated” has been replaced with “investigated and compared” to more accurately reflect that our study’s purpose was to assess and compare the effects of the two AI strategies.

We believe this change clarifies the positioning of our research and are grateful for the opportunity to improve the manuscript. Thank you for your valuable suggestion.
