# Supplementary material for: Deepening ideas vs. exploring new ones: AI strategy effects in human-AI creative collaboration
Source: PLoS One. 2026 Jan 7;21(1):e0340449. doi: 10.1371/journal.pone.0340449 (PMC12779158; doi:10.1371/journal.pone.0340449)
Supplement: Attachment — Submitted filename: Response to Reviewers251214.docx [file pone.0340449.s005.docx]

Manuscript ID: PONE-D-25-40481

Title: Deepening Ideas vs. Exploring New Ones: AI Strategy Effects in Human-AI Creative Collaboration

Journal: PLOS ONE

Dear Dr. Piwowar-Sulej and Reviewers,

Thank you for the opportunity to submit a revised version of our manuscript. We appreciate your careful consideration and constructive guidance. We have revised the manuscript to address the points raised by the Academic Editor. Below we provide a point-by-point response. For ease of checking, page and line numbers refer to the revised clean manuscript. We have also uploaded (i) the Response to Reviewers, (ii) the Revised Manuscript with Track Changes, and (iii) the Clean Manuscript.

Sincerely,

Kazuki Komura

### *Journal Requirements*

1. If the reviewer comments include a recommendation to cite specific previously published works, please review and evaluate these publications to determine whether they are relevant and should be cited. There is no requirement to cite these works unless the editor has indicated otherwise.

We reviewed the publication suggested by the Academic Editor and evaluated its relevance to our manuscript. As it provides a useful conceptual foundation for discussing competency development in human–technology interactions, we have cited it in the revised manuscript where we discuss the competency-development perspective in human–AI collaboration (clean manuscript: p.19, line 705).

2. Please review your reference list to ensure that it is complete and correct. If you have cited papers that have been retracted, please include the rationale for doing so in the manuscript text, or remove these references and replace them with relevant current references. Any changes to the reference list should be mentioned in the rebuttal letter that accompanies your revised manuscript. If you need to cite a retracted article, indicate the article’s retracted status in the References list and also include a citation and full reference for the retraction notice.

We reviewed the reference list for completeness and accuracy and confirmed that no retracted items are cited. We also updated the reference list to reflect the newly added citation noted above, and this change is described in this rebuttal letter.

### ***Response to* Academic Editor**

We sincerely thank the editor for the time, careful reading, and constructive comments. We have revised the manuscript accordingly and indicate specific changes and locations below.

***“The manuscript presents interesting and timely insights into human–AI collaboration; however, to further strengthen the contribution, the authors should more explicitly address the role of human–AI collaboration in the development of human competencies. This aspect carries both theoretical and practical significance and would enrich the discussion on how AI can support—not only complement—human creative and cognitive capabilities. I recommend that the authors incorporate relevant perspectives from recent research on competence development in human–technology interactions . The following article offers a useful conceptual foundation*** [***https://doi.org/10.1108/JOCM-10-2023-0426***](https://doi.org/10.1108/JOCM-10-2023-0426)***. Integrating this dimension would enhance the theoretical depth of the manuscript and clarify the practical implications of designing AI systems that not only collaborate with humans but also help them learn, grow, and expand their capabilities. Please note that citation of this is entirely optional but we hope it will be helpful overall****.”*

We agree that this dimension would strengthen the contribution. To address this point while keeping our claims appropriately cautious (since our study does not directly measure skill acquisition), we added a concise discussion that explicitly connects our empirical findings to the possibility that certain forms of human–AI interaction may support learning and competency development over time.

Concretely, we added a paragraph at the end of the “Convergent Strategies for Divergent Goals” subsection in the Discussion (clean manuscript: p.19, lines 697–707) to (i) link our empirical findings to the possibility that repeated human–AI interaction in creative tasks may foster collaboration-relevant competencies over time (e.g., learning to engage with AI contributions more effectively as users become familiar with the AI’s interaction pattern), (ii) connect this perspective to our observed results (e.g., higher trust ratings and greater adoption of AI ideas under the deepening strategy), and (iii) clearly state the limitation that competency development was not directly measured and should be examined in future longitudinal work. In this added text, we cite the suggested work as a conceptual foundation for competency development in human–technology interactions (added as Ref. [56]).

***“In the Practical Implications section, you stated that “the empirical results of this study provide concrete guidelines for the design of human–AI creative collaboration systems.” However, the manuscript does not offer specific, actionable guidance for different types of organizations. Please clarify which industries, sectors, or organizational contexts could directly benefit from your findings”***

We thank the Academic Editor for this important point. We expanded the Practical Implications section to provide specific, actionable guidance for organizational contexts that could directly benefit from our findings (clean manuscript: p.20, lines 742–763). Specifically, we now identify representative sectors/organizational settings (e.g., marketing/advertising ideation, product development/innovation teams, and educational settings for AI literacy and creative thinking) and explain how the observed strengths of the deepening strategy translate into practical design and deployment guidance. We also adopt a cautious tone to avoid overgeneralization beyond our experimental setting.

In addition, we make the added guidance explicit by summarizing the three design principles (trust-building, short-term adoption of AI ideas, and the link between understanding AI behavior and collaboration effectiveness) and by describing implementation guidance such as starting with deepening-oriented systems to establish trust before introducing broadening strategies once familiarity is established.

### Response to Reviewer #2

We sincerely thank Reviewer #2 for their positive evaluation and for confirming that all prior comments have been addressed. No additional changes were requested by Reviewer #2 in this round, and our revisions focused on the Academic Editor’s two points above.
